# Factors influencing use of conventional and traditional Korean medicine-based health services: a nationwide cross-sectional study

**DOI:** 10.1186/s12906-022-03641-x

**Published:** 2022-06-20

**Authors:** Yui Sasaki, Jeong-Su Park, Sunju Park, Chunhoo Cheon, Yong-Cheol Shin, Seong-Gyu Ko, Bo-Hyoung Jang

**Affiliations:** 1 Policy Promotion Department, Institute for Health Economics and Policy, Association for Health Economics Research and Social Insurance and Welfare, 1-5-11, Nishi-Shimbashi, Minato-ku, Tokyo, 105-0003 Japan; 2 Department of Preventive Medicine, College of Korean Medicine, Semyung University, 65 Semyeong-ro, Jecheon, Chungcheongbuk-do 27136 Republic of Korea; 3 Department of Preventive Medicine, College of Korean Medicine, Daejeon University, 62 Daehak-ro, Dong-gu, Daejeon, 34520 Republic of Korea; 4 Department of Preventive Medicine, College of Korean Medicine, Kyung Hee University, 26 Kyungheedae-ro, Dongdaemun-gu, Seoul, 02447 Republic of Korea

**Keywords:** Andersen’s behavioral model, Healthcare system, Health service use, Conventional medicine, Traditional Korean medicine, Survey on the Experience with Healthcare Services

## Abstract

**Background:**

In Korea, conventional medicine (CM) and traditional Korean medicine (KM) are run as a dual healthcare system; however, the backgrounds and characteristics of the users of both medical services have not yet been compared. This study aimed to identify the differences in factors determining the use of CM and KM health services.

**Methods:**

A secondary data analysis of a nationwide cross-sectional survey was conducted in this study. The Survey on the Experience with Healthcare Services 2017 asked participants about their most recent outpatient visit to a health service. Initially, a descriptive analysis was performed on respondents who visited the CM or KM health service in the last 12 months. Then, logistic regression analysis using Andersen’s behavioral model was performed, to identify the factors affecting health service selection, by classifying demographic variables into predisposing, enabling, and need factors. Respondents who replied they did not frequently use CM/KM and those with missing data were excluded.

**Results:**

Of the total 11,098 respondents, 7,116 (64.1%) reported to have used CM/KM: 2,034 (18.3%), 4,475 (40.3%), and 607 (5.5%) for hospital CM, clinic CM, and KM, respectively. In logistic regression analysis, of the 2,723 (24.5%) respondents analyzed, 822 (7.4%) went to a hospital, 1,689 (15.2%) to a clinic, and 212 (1.9%) opted for KM service. Respondents with a higher number of chronic diseases were less likely to use KM (one disease, odds ratio: 0.52, 95% confidence interval: 0.36–0.76; two diseases: 0.51, 0.31–0.85; three to five diseases: 0.26, 0.10–0.69). Respondents with a high income were likely to go to the hospital (4Q vs. 1Q: 1.92, 1.35–2.72) and less likely to go to the clinic (4Q vs. 1Q: 0.49, 0.35–0.68).

**Conclusions:**

Significant differences were observed on the enabling factor (income) for CM and need factors (number of chronic diseases) for KM. Our analysis suggests that through the healthcare policy, we should consider stratifying user backgrounds and needs for each medical service.

**Supplementary Information:**

The online version contains supplementary material available at 10.1186/s12906-022-03641-x.

## Background

Unlike in most countries, the medical system in Korea is dichotomized into conventional medicine (CM) and traditional Korean medicine (KM) practices. Hence, both CM and KM use different systems for education, licensing, hospital facilities, insurance, and legal matters. Therefore, patients in Korea can choose between two medical services.

The KM system provides services such as herbal medicine, acupuncture, cupping, and heat therapy under the national health insurance (NHI) system. A 2017 survey among Korean citizens revealed that 73.8% of the respondents had used KM [1]. Among them, 90.2%, 53%, and 49.1% had received acupuncture, cupping, and moxibustion treatments, respectively [1]. In 2017, the total medical fees associated with outpatient KM services totaled 2.286 trillion Korean won (KRW), and 103 million requests for KM services were made, accounting for 5.1% and 7.4% of all outpatient fees and requests, respectively [2]. These statistics indicate that people in Korea widely used KM. Previous studies have reported an association between the use of KM services and demographic factors such as gender, age, residence area, and household income [3–5]. However, to the best of our knowledge, no previous study has compared factors influencing the use of CM and KM. Therefore, this study aimed to clarify the factors influencing the use of each medical service, which can support the development of healthcare policies.

The Survey on the Experience with Healthcare conducted by the Ministry of Health and Welfare (MOHW) and Korea Institute for Health and Social Affairs (KIHASA) was conducted in 2017. Because the nationwide survey is for the general population rather than patients, the respective characteristics of CM and KM use can be compared. Therefore, this study aimed (1) to describe the frequency (%) of using CM- and KM-based services and (2) to clarify the factors associated with their use. To select these factors, Andersen’s behavioral model, which has been widely used to analyze factors influencing medical services, was applied as described in a previous study [6].

## Methods

### Study design

This study was designed as a secondary analysis of the population-based data from the Survey on the Experience with Healthcare Services 2017, a nationwide cross-sectional survey.

### Data source

During the second half of 2017, the MOHW and KIHASA replaced a cross-sectional patient survey that had been used for several decades with the newly formed “Survey on the Experience with Healthcare.” This new survey primarily aimed to identify the disease and disability prevalence and actual use of medical services and to provide a basis for establishing a standard healthcare policy [7]. The survey addressed the following issues: medical services, healthcare experiences (services provided by physicians, nurses, and medical facilities and safety and waiting time), satisfaction levels of out- and inpatients, healthcare system perceptions, and health status.

The survey was conducted by a trained investigator via face-to-face interviews of household members aged ≥ 15 years. Regarding the sampling method, 5,000 households were selected as samples from the Population and Housing Census [8]; namely, the total target population size was 19,475,340 households (42,798,956 individuals) [7].

### Sampling procedure

Of the 11,098 total survey respondents, 8,057 (72.6%) had visited any healthcare service (hospital, clinic, KM hospital/clinic, dental, public health, and others) as an outpatient between January 2017 and the time of the survey, whereas 3,041 (27.4%) had not used such services. Respondents recalled their most recent visit to a healthcare service during the survey period. Initially, 7,116 respondents who visited a hospital, clinic, or KM were included in the descriptive analysis and comparison of results, excluding those who visited for dental (754), public health (113), and other (74) concerns. The definition of each healthcare facility is provided in Supplementary Table 1 [7]. Given that the survey included questions about the health service usage in the last 12 months, respondents who happened to use CM or KM by chance were also included. However, they should have been excluded as much as possible to identify the specific factors of each health service. Therefore, the present study only included respondents who have been using healthcare services frequently (repeat users in the same medical facility) in the logistic regression analysis. Respondents with missing data for Andersen’s behavioral model were excluded (Supplementary Fig. 1).

### Dependent variables in the logistic regression analysis

The use of CM or KM healthcare services (yes/no) since January 2017 was set as a dichotomous-dependent variable.

### Independent variable in the logistic regression analysis

Andersen’s behavioral models of predisposing, enabling, and need factors were used in our logistic regression analysis (enter method; Table 1). This behavioral model was initially developed in 1968 to describe factors associated with the use of health services [6]. *Predisposing factors* included gender, age, educational level, and occupation; *enabling factors* included the monthly household income, area of residence, and health insurance type; and *need factors* included the number of chronic diseases and general health status.

**Table 1:** Definition of independent variables

Factors	Variable name	Subcategory
**Predisposing factors**	Gender	male/female
	Age	≤ 30’s/40’s/50’s/60’s/≥ 70’s
	Education completed	elementary (≤ 6y)/middle-high (7-12y)/university (≥ 12y)
	Occupation	office workers/self-employed, employer/housewives/students/unemployed/others
**Enabling factors**	Household income (monthly)	Quartile 1; lowest (KRW < 1,500,000)/ Quartile 2 (KRW 1,500,000 ≤ - < 3,500,000)/ Quartile 3 (KRW 3,500,000 ≤ - < 5,500,000)/ Quartile 4; highest (KRW ≥ 5,500,000)
	Area of residence	urban/rural
	Health insurance type	national health insurance/medical aid
**Need factors**	No. of chronic diseases ^a^	0/1/2/≥ 3
	General health status	very good/good/acceptable/poor/very poor

*Abbreviations*: *KRW* Korean Won

^a^hypertension/diabetes/hyperlipidemia/arthropathies/tuberculosis/ischemic heart disease/cerebrovascular disease/others

### Statistical analysis

The present comparative analysis of healthcare service use was performed in three stages. First, a descriptive analysis was performed to determine the frequency (%) of distributions using chi-squared tests, followed by a descriptive analysis to determine the reason for using CM/KM healthcare services. Next, logistic regression analysis was used to identify factors influencing their use, including predisposing factors, enabling factors, and need factors. The results are reported as odds ratios (ORs) with 95% confidence intervals (CIs) relative to the first subgroup as a reference. All statistical analyses were conducted using the Statistical Package for the Social Sciences version 25.0 (IBM Corp, Armonk, NY, USA). A *p*-value of < 0.05 was considered statistically significant.

## Results

### Demographics

Of the total 7,116 respondents who visited the CM or KM health service in the last 12 months: 2,034 for hospital CM, 4,475 for clinic CM, and 607 for KM, respectively. Table 2 presents the demographic characteristics of the respondents stratified by CM and KM. Of the total 7,116 respondents, 57.4% were women, 55.4% completed middle-high (7–12 years) educational level, 70.8% resided in urban areas, and 97.2% were under the NHI. The highest rate of respondents’ ages in each healthcare facility was as follows: hospital, 60–69 years (23.2%); clinic, ≤ 39 years (28.5%); and KM, 50–59 years (26.2%). Regarding occupation, 3–10 of all healthcare users were office workers. The household income was presented by quartile; 52.4% of all respondents had missing values, and approximately 16% of all healthcare users were in Quartile 2.

**Table 2:** Demographic characteristics by healthcare services (*n* = 7,116)

Characteristics	n (%)				*p*-value^a^
	**Total** **(** ***n*** ** = 7,116)**	**Hospital** **(** ***n*** ** = 2,034)**	**Clinic** **(** ***n*** ** = 4,475)**	**KM** **(** ***n*** ** = 607)**	
**Gender**					< 0.0001
** Male**	3,030 (42.6)	932 (45.8)	1,872 (41.8)	226 (37.2)	
** Female**	4,086 (57.4)	1,102 (54.2)	2,603 (58.2)	381 (62.8)	
**Age**					< 0.0001
** ≤ 39**	1,747 (24.5)	392 (19.3)	1,275 (28.5)	80 (13.2)	
** 40–49**	1,101 (15.5)	270 (13.3)	727 (16.2)	104 (17.1)	
** 50–59**	1,633 (22.9)	461 (22.7)	1,013 (22.6)	159 (26.2)	
** 60–69**	1,381 (19.4)	472 (23.2)	754 (16.8)	155 (25.5)	
** ≥ 70**	1,254 (17.7)	439 (21.6)	706 (15.8)	109 (18.0)	
**Education completed**					< 0.0001
** Elementary (≤ 6y)**	1,034 (14.5)	337 (16.6)	626 (14.0)	71 (11.7)	
** Middle-High (7-12y)**	3,941 (55.4)	1,141 (56.1)	2,432 (54.3)	368 (60.6)	
** University (**≥ **12y)**	2,141 (30.1)	556 (27.3)	1,417 (31.7)	168 (27.7)	
**Occupation**^**b**^					< 0.0001
** Office workers**	2,444 (34.4)	638 (31.4)	1,599 (35.8)	207 (34.1)	
** Self-employed, Employer**	1,322 (18.6)	392 (19.3)	804 (18.0)	126 (20.8)	
** Housewives**	1,904 (26.8)	533 (26.2)	1,176 (26.3)	195 (32.1)	
** Students**	503 (7.1)	96 (4.7)	399 (8.9)	8 (1.3)	
** Unemployed**	757 (10.7)	296 (14.6)	400 (9.0)	61 (10.0)	
** Others**	172 (2.4)	75 (3.7)	87 (1.9)	10 (1.6)	
*** Missing***	14 (0.4)	4 (0.4)	10 (0.4)	0	
**Household income** ^c^					0.002
** Quartile 1**	866 (12.2)	281 (13.8)	517 (11.6)	68 (11.2)	
** Quartile 2**	1,189 (16.7)	334 (16.4)	755 (16.9)	100 (16.5)	
** Quartile 3**	870 (12.2)	253 (12.4)	558 (12.5)	59 (9.7)	
** Quartile 4**	458 (6.4)	163 (8.0)	245 (5.5)	50 (8.2)	
*** Missing***	3,733 (52.4)	1,003 (49.3)	2,400 (53.6)	330 (54.4)	
**Area of residence**					0.276
** Urban**	5,040 (70.8)	1,449 (71.2)	3,146 (70.3)	445 (73.3)	
** Rural**	2,076 (29.2)	585 (28.8)	1,329 (29.7)	162 (26.7)	
**Health insurance type** ^d^					< 0.0001
** NHI**	6,916 (97.2)	1,940 (95.4)	4,378 (97.8)	598 (98.5)	
** Medical aid**	181 (2.5)	86 (4.2)	88 (2.0)	7 (1.2)	
*** Missing***	19 (0.3)	8 (0.4)	9 (0.2)	2 (0.3)	

*Abbreviations: KM *Korean Medicine, *NHI *national health insurance

^a^Statistical analysis: Chi-square test was used to compare categorical variable (*p*-value < 0.05)

^b^ 0.2% of data missing

^c^ 51.8% of data missing

^d^ 0.3% of data missing

### Reasons cited for healthcare service utilization

**Fig. 1:**
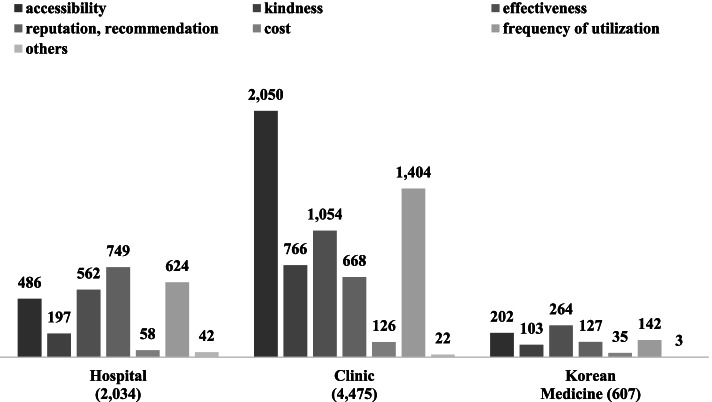
Bar chart for the reasons that visited health service (multiple answers, respectively)

The reason for using a certain healthcare service was also asked in the survey, with accessibility, kindness, effectiveness, reputation/recommendation, cost, frequency of utilization, and others (multiple answers) as options. Results of descriptive statistics were presented to identify differences in their reasons for choosing CM and KM. The following reasons were most commonly cited for the use of each type of healthcare service: reputation/recommendation for hospital facilities (749 [36.8%] of 2,034 respondents); access to a clinic (2,050 [45.8%] of 4,475); effectiveness for KM services (264 [43.5%] of 607). The second most common reasons were: frequency of utilization for a hospital (624 [30.7%] of 2,034) and clinic (1,404 [31.4%] of 4,475), as well as access to a KM (202 [33.3%] of 607) (Fig. 1).

### Factors influencing the use of CM and KM

Of the total 2,723 respondents who replied “frequently using,” 822/2,034 (40.4%), 1,689/4,475 (37.7%), and 212/607 (34.9%) went for a hospital, clinic, and opted for KM, respectively. Table 3 compares the results of logistical regression analyses by hospital, clinic, and KM use. In the clinic group, the use significantly decreased in respondents aged ≥ 60 years (OR, 0.68; 95% CI, 0.47–0.97 for 60–69; and OR, 0.64; 95% CI, 0.42–0.97 > 70 years) and in those who had completed education (OR, 0.61; 95% CI, 0.46–0.81 for 7–12 years; and OR, 0.67; 95% CI, 0.47–0.95 for ≥ 12 years). The analysis yielded a higher OR for hospital use (OR, 1.40; 95% CI, 1.02–1.90 in Quartile 3 and OR, 1.92; 95% CI, 1.35–2.72 in Quartile 4) among high-income respondents than low-income respondents, whereas, this association was not observed in those using the clinic (except in Quartile 4: OR, 0.49; 95% CI, 0.35–0.68) and KM services. In both CM (hospital) and KM groups, the ORs increased as the general health status worsened, whereas in the KM group, the use significantly decreased as the number of chronic diseases increased (OR, 0.52; 95% CI, 0.36–0.77 for 1 disease; OR, 0.51; 95% CI, 0.31–0.85 for 2; and OR, 0.26; 95% CI, 0.10–0.69 for 3–5 diseases). No significant differences in gender, occupation, and area of residence were observed among the groups.

**Table 3:** Influential factors associated with use of a healthcare service in logistic regression analysis (*n* = 2,723)

Variables	OR (95% CI) ^a^		
	**Hospital (** ***n*** ** = 822)**	**Clinic (** ***n*** ** = 1,689)**	**KM (** ***n*** ** = 212)**
**Gender (Ref: Male)**			
** Female**	0.91 (0.72–1.14)	1.05 (0.81–1.25)	1.30 (0.90–1.87)
**Age (Ref: ≤ 39)**			
** 40–49**	0.78 (0.53–1.12)	1.30 (0.92–1.84)	0.86 (0.48–1.54)
** 50–59**	1.01 (0.71–1.44)	1.07 (0.77–1.48)	0.78 (0.44–1.39)
** 60–69**	1.39 (0.94–2.05)	**0.68 (0.47–0.97)**	1.32 (0.71–2.45)
** ≥ 70**	1.50 (0.95–2.35)	**0.64 (0.42–0.97)**	1.30 (0.62–2.71)
**Education completed (Ref: Elementary (≤ 6y))**			
** Middle High (7-12y)**	**1.43 (1.07–1.92)**	**0.61 (0.46–0.81)**	**1.71 (1.02–2.88)**
** University (**≥ **12y)**	1.28 (0.88–1.85)	**0.67 (0.47–0.95)**	1.78 (0.93–3.40)
**Occupation (Ref: Office workers)**			
** Self-employed, Employer**	0.82 (0.65–1.03)	1.14 (0.92–1.42)	1.14 (0.77–1.67)
** Housewives**	0.79 (0.56–1.11)	1.30 (0.95–1.79)	0.84 (0.48–1.48)
** Students**	0.96 (0.25–3.68)	1.57 (0.41–5.96)	-
** Unemployed**	0.99 (0.74–1.35)	0.97 (0.72–1.29)	1.09 (0.65–1.84)
** Others**	1.49 (0.86–2.60)	0.63 (0.37–1.08)	1.31 (0.52–3.28)
**Household income (Ref: Quartile 1)**			
** Quartile 2**	1.14 (0.87–1.49)	0.90 (0.70–1.16)	0.94 (0.60–1.46)
** Quartile 3**	**1.40 (1.02–1.90)**	0.79 (0.59–1.05)	0.82 (0.48–1.39)
** Quartile 4**	**1.92 (1.35–2.72)**	**0.49 (0.35–0.68)**	1.44 (0.82–2.53)
**Area of residence (Ref: Urban)**			
** Rural**	0.84 (0.68–1.02)	1.19 (0.98–1.44)	0.97 (0.69–1.37)
**Health insurance type (Ref: NHI)**			
** Medical Aid**	**1.94 (1.23–3.05)**	0.68 (0.44–1.07)	0.26 (0.06–1.09)
**No. of chronic diseases** ^**b**^ **(Ref: 0)**			
** 1**	1.20 (0.96–1.50)	1.05 (0.85–1.29)	**0.52 (0.36–0.76)**
** 2**	1.12 (0.83–1.51)	1.13 (0.85–1.49)	**0.51 (0.31–0.85)**
** 3–5**	1.49 (0.98–2.27)	0.98 (0.65–1.48)	**0.26 (0.10–0.69)**
**General health status (Ref: Very Good)**			
** Good**	1.10 (0.76–1.60)	0.79 (0.55–1.11)	1.74 (0.85–3.54)
** Acceptable**	1.23 (0.83–1.83)	**0.64 (0.44–0.92)**	**2.44 (1.16–5.10)**
** Poor**	**2.11 (1.34–3.30)**	**0.34 (0.22–0.53)**	**3.73 (1.62–8.56)**
** Very Poor**	**3.37 (1.50–7.55)**	**0.30 (0.14–0.67)**	-

*Abbreviations: KM *Korean Medicine, *OR *odds ratio, *CI *confidence interval, *NHI *national health insurance

^a^ Adjusted for: predisposing, enabling and need factors (gender, age, education completed, occupation, household income, area of residence, health insurance type, No. of chronic diseases, and general health status)

^b^ hypertension/diabetes/hyperlipidemia/arthropathies/tuberculosis/ischemic heart disease/cerebrovascular disease/other

## Discussion

This secondary analysis of data from the Survey on the Experience with Healthcare investigated the use of healthcare services in Korea and the factors influencing the behaviors via descriptive statistics and logistic regression. An analysis of 2,723 respondents who regularly used CM and KM demonstrated significant differences in factors influencing the use of CM and KM.

In our descriptive analysis, women aged > 50 who lived in urban areas accounted for more than half of the respondents in both CM and KM groups. These results were largely consistent with previous studies [3–5, 9]. However, the logistic regression analysis showed that high income was not associated with the use of KM, unlike in a previous study [5]; in addition, a negative association was observed between the number of chronic diseases and KM use. A previous cross-sectional study of Korean inpatients reported that 79.0% (334/423) of those with multiple chronic diseases used complementary and alternative medicine (CAM) with CM; of these, 91.3% reported having used KM [10]. A comparison of our findings and those of previous studies indicate the presence of various contradictions, such as the characteristics of KM users [4, 5, 10].

The above-described contradictions may be attributed to the methodology, particularly sample selection. Our inclusion criteria included only respondents who frequently used the same medical facility. A 2017 survey of KM uses that particularly targeted outpatients demonstrated that 26.4%, 30.7%, 19.4%, and 23.5% of the respondents had used services 1–3, 4–7, 8–12, and ≥ 13 times, respectively, in the past 12 months (mean: 11.6 times). Additionally, 93.3% replied that they had sought KM services to treat an illness [11]. These results imply the following motivations of KM service use: (1) acute disease, (2) chronic disease requiring a long treatment period, and (3) continued treatment for purposes other than the treatment of a primary disease. A survey that compared the provision for CAM based on medical providers reported that 26.7%, 29.6%, and 38.3% cited acute illness, long-term illness, and improved well-being, respectively, as their reasons for KM use, whereas 35.1%, 30.0%, and 27.7% cited these factors, respectively, as their reasons for CM use [12]. The Survey on the Experience with Healthcare Services did not consider whether respondents received medical treatment covered by the NHI. In our study, although KM users most commonly cited effectiveness as the reason for opting for KM (Fig. 1), determining whether this reason was for disease treatment or well-being improvement is impossible. If KM users reported effectiveness in terms of well-being improvement, the number of chronic diseases was possibly low because they were not aimed at disease treatment. Users who frequently use KM services tend to choose KM over CM from the perspective of prevention rather than treatment.

CM users showed different results for the hospital- and clinic-groups. Our study indicates that people aged > 60, highly educated, and high income were statistically less likely to visit the clinic. These results were largely consistent with those of a previous study of factors that indicated that patients were more likely to visit the hospital [13]. Moreover, a hospital user might be included under Medical Aid Beneficiaries [14, 15], and older-adults prefer public health clinics over private clinics [16].

With regard to needs factors on CM, a number of chronic diseases were not statistically significant factors in our study. As described above, not only KM users but also certain CM users stated that they wished to improve their well-being, which was their reason for use. Given that the survey is for the general population rather than patients, needs factors do not necessarily imply a need for treatment is required. Hence, considering the characteristics of health service use for the general population, results will vary depending on whether the purpose is to treat a disease or improve well-being (covered by the NHI or not) and whether each service is for long- or short-term use (frequently used or not).

Differences in data sources and factors included in Andersen’s behavioral model could explain differences between our study and previous studies. The Korea Health Panel Study data were used in previous comparisons between CM and KM use [17]. Although the survey included questions about marital status and private health insurance, it did not address constant use; accordingly, differences between surveys would likely affect the outcomes of analyses. Moreover, Andersen’s behavioral model has evolved with improvements over time (updates in 1975, 1995, and 2003). Therefore, researchers can choose a model for analysis based on the process. Previous studies of KM mainly applied the 1975 or 1995 model because data sources limited the factors available for analysis. Particularly, challenging to investigate external environmental characteristics (e.g., healthcare systems) [6]. A previous study indicated that socioeconomic status likely determined the decisions of CM and KM use [4]. Further studies are needed to analyze and compare healthcare services based on these aspects.

Several limitations of this study should be acknowledged. First, a risk of nonresponse bias may exist. Second, the risk of recall bias may also occur due to the retrospective survey design. Third, the questionnaire inquired about the most recent use of healthcare services since January 2017, and the sample would have included respondents who used more than one healthcare service. In other words, multiple-healthcare users were not controlled and did not strictly compare CM and KM. Moreover, the number of KM in our study was relatively small. However, this survey benefited from the use of the same questionnaire to determine the current state of use of both systems.

## Conclusions

Our secondary data analysis from a nationwide cross-sectional survey compared KM and CM use in the Korean population. A logistic regression analysis based on Andersen’s behavioral model indicates that people with a higher number of chronic diseases were statistically less likely to use KM. No similar association was observed in CM use. Meanwhile, people with high incomes were statistically likely to use hospitals but were less likely to use clinics. Factors influencing the use of CM and KM were found to be different. These findings suggest that the different characteristics and needs of users opting for CM and KM should be considered in formulating healthcare policies.

## Supplementary Information

**Additional file 1: Supplementary Table 1.** Definitions for Healthcare Services in this Study. **Supplementary Fig. 1. ** Flowchart of the Selection Process.

## Data Availability

Datasets generated in the current study are available in the Korea Institute for Health and Social Affairs repository, https://data.kihasa.re.kr/kihasa/main.html.

## References

[1] Ministry of Health and Welfare, National Development Institute of Korean Medicine. Report on Usage and Consumption of Korean Medicine 2017; Basic report (for citizens). https://www.koms.or.kr/board/researchReport/view.do?post_no=45&menu_no=21. Accessed 23 Jan 2022. [In Korean]

[2] Korea Institute of Oriental Medicine, Korea Oriental Medicine Association, Korean Traditional Medicine Foundation, Graduate School of Oriental Medicine, Pusan National University. 2018 Year Book of Traditional Korean Medicine. Daejeon Korea. https://www.kiom.re.kr/brdartcl/boardarticleView.do?menu_nix=WUNNW2Aq&brd_id=BDIDX_o9YEVvNb40b134N1Rt17aq&cont_idx=9. Accessed 23 Jan 2022. [In Korean]

[3] Kim D, Lim B, Kim C (2015). Relationship between patient satisfaction with medical doctors and the use of traditional Korean medicine in Korea. BMC Complement Altern Med.

[4] Park JE, Kwon S (2011). Determinants of the utilization of oriental medical services by the elderly. J Korean Oriental Med.

[5] Choi JH, Kang S, You CH, Kwon YD (2015). The determinants of choosing traditional Korean medicine or conventional medicine: findings from the Korea Health Panel. Evid Based Complement Alternat Med.

[6] Andersen RM (1995). Revisiting the behavioral model and access to medical care: does it matter?. J Health Soc Behav.

[7] Ministry of Health and Welfare. Survey on the Experience with Healthcare Services 2017. http://www.mohw.go.kr/react/jb/sjb030301vw.jsp?PAR_MENU_ID=03&MENU_ID=032901&CONT_SEQ=343794. Accessed 23 Jan 2022. [In Korean]

[8] Statistics Korea, Population Census, Overview. http://kostat.go.kr/portal/eng/surveyOutline/8/5/index.static. Accessed 23 Jan 2022.

[9] Park MJ, Kwon S (2014). Socioeconomic determinants of Korean medicine ambulatory services: comparing panel fixed effect model with pooled ordinary least square. Health Policy Manag.

[10] Choi B, Han D, Na S, Lim B (2017). Factors related to the parallel use of complementary and alternative medicine with conventional medicine among patients with chronic conditions in South Korea. Integr Med Res.

[11] Ministry of Health and Welfare, National Development Institute of Korean Medicine. Korean Medicine Utilization Survey 2017; Basic report for user. https://www.mohw.go.kr/react/gm/sgm0704vw.jsp?PAR_MENU_ID=13&MENU_ID=1304080305&page=1&CONT_SEQ=356996&PAR_CONT_SEQ=355693. Accessed 23 Jan 2022. [In Korean]

[12] Lee JA, Sasaki Y, Arai I, Go HY, Park S, Yukawa K (2018). An assessment of the use of complementary and alternative medicine by Korean people using an adapted version of the standardized international questionnaire (I-CAM-QK): a cross-sectional study of an internet survey. BMC Complement Altern Med.

[13] Lee JH, Choi YJ, Lee SH, Sung NJ, Kim SY, Hong JY (2013). Association of the length of doctor-patient relationship with primary care quality in seven family practices in Korea. J Korean Med Sci.

[14] Kim JH, Lee SG, Lee KS, Jang SI, Cho KH, Park EC (2016). Impact of health insurance status changes on healthcare utilisation patterns: a longitudinal cohort study in South Korea. BMJ Open..

[15] Lee DW, Jang J, Choi DW, Jang SI, Park EC (2020). The effect of shifting medical coverage from National Health Insurance to Medical Aid type I and type II on health care utilization and out-of-pocket spending in South Korea. BMC Health Serv Res.

[16] Kim AM, Cho S, Kim HJ, Jo MW, Eun SJ, Lee JY (2018). Rethinking the Role of the Public Health Clinic: Comparison of Outpatient Utilization in the Public Health Clinics and Private Clinics in Korea. Int J Environ Res Public Health.

[17] Korea Institute for Health and Social Affairs, National Health Insurance Service: Korea Health Panel Study. https://www.khp.re.kr:444/eng/main.do. Accessed 23 Jan 2022. [In Korean]

