# Supplementary material for: Factors influencing use of conventional and traditional Korean medicine-based health services: a nationwide cross-sectional study
Source: BMC Complement Med Ther. 2022 Jun 20;22:162. doi: 10.1186/s12906-022-03641-x (PMC9208109; doi:10.1186/s12906-022-03641-x)
Supplement: Supplementary file 1 — Additional file 1: Supplementary Table 1. Definitions for Healthcare Services in this Study. Supplementary Fig. 1. Flowchart of the Selection Process. [file 12906_2022_3641_MOESM1_ESM.docx]

**Supplementary table 1:**

**Definitions for Healthcare Services in this Study**

| **Healthcare Service** | | **Definition** |
| --- | --- | --- |
| **Conventional Medicine** | **Hospital** | Medical institutions (including general hospitals, and tertiary hospitals) can accommodate 30 or more inpatients. |
|  | **Clinic** | Medical institutions can accommodate less than 30 inpatients. |
| **Korean Medicine** | | Medical institutions that only provide traditional Korean medical treatment . |

Note: Outpatients in this survey did not include for the following purposes: medical examination, plastic surgery, beauty treatments, and obesity management.

**Supplementary figure 1:**

**Flowchart of the Selection Process**

All participants for the Survey on the Experience with Healthcare Services 2017
(n = 11,098)

NOT used healthcare services (n = 3,041)
Used the following healthcare services (n = 941)
- Dental (n = 754)
- Public health (n = 113)
- Others (n = 74)

Used the following healthcare services (n = 7,116)
- Hospital (n = 2,034)
- Clinic (n = 4,475)
- Korean Medicine (n = 607)

NOT using frequently the following healthcare services (n = 1,423)
- Hospital (n = 458)
- Clinic (n = 849)
- Korean Medicine (n = 116)

Using frequently the following healthcare services
(n = 5,693)
- Hospital (n = 1,576)
- Clinic (n = 3,626)
- Korean Medicine (n = 491)

NOT replied demographic factors and factors for Andersen’s behavioral model (n = 2,970)
- Hospital (n = 754)
- Clinic (n = 1,937)
- Korean Medicine (n = 279)

Eligible participants for logistic regression model
(n = 2,723)
- Hospital (n = 822)
- Clinic (n = 1,689)
- Korean Medicine (n = 212)
